# Multilevel Transcriptomic Association Analysis Reveals Key Genes and Potential Mechanisms in Endometrial, Ovarian, and Cervical Cancers

**DOI:** 10.1007/s43032-025-02010-6

**Published:** 2025-11-13

**Authors:** Liang Liu, Xinghao Zhao

**Affiliations:** 1 Henan Provincial People’s Hospital, People’s Hospital of Zhengzhou University, Zhengzhou, 450003 Henan China; 2 Department of International Medical Center, Henan Provincial People’s Hospital, People’s Hospital of Zhengzhou University, No.7, Weiwu Road, Jinshui District, Zhengzhou, 450003 Henan China; 3 Department of Gynecology, Henan Provincial People’s Hospital, People’s Hospital of Zhengzhou University, No.7, Weiwu Road, Jinshui District, Zhengzhou, 450003 Henan China

**Keywords:** Ovarian cancer, Cervical cancer, Endometrial cancer, SPX, Transcriptomic analysis

## Abstract

Objective: This study aims to investigate the genetic associations of endometrial cancer (EC), ovarian cancer (OC), and cervical cancer (CC), identify potential key genes using multiple genomic analysis approaches, and analyze their roles in cancer development. Methods: We integrated large-scale genome-wide association study (GWAS) data from Jiang L et al. and Zhou W et al., combined with blood eQTL data. We employed S-PrediXcan, SMR, and mBAT-combo to assess gene associations with EC, OC, and CC. Additionally, RNA sequencing data were used to analyze the expression levels of key genes across different tissues, followed by functional enrichment analysis to explore their potential biological functions. Results: Through S-PrediXcan, SMR, and mBAT-combo analyses, we identified 690, 620, and 624 genes associated with OC, CC, and EC, respectively. Among them, 79, 59, and 61 genes were consistently significant across all three methods, suggesting their crucial roles in cancer development. Furthermore, we identified multiple comorbidity-related genes, including SPX, which exhibited a negative association with OC, CC, and EC. Transcriptomic analysis revealed that 26 key genes displayed significant expression differences between tumor and normal tissues. Functional enrichment analysis further identified the molecular pathways potentially involved. Conclusion: This study identified a set of key genes associated with EC, OC, and CC and suggested that SPX may play a protective role in cancer development. The integration of multilevel genetic and transcriptomic analyses provides new insights into the molecular mechanisms underlying gynecological malignancies and offers potential biomarkers for future precision medicine research.

**Supplementary Information:**

The online version contains supplementary material available at 10.1007/s43032-025-02010-6.

## Introduction

Gynecologic malignancies are among the major cancer types threatening women’s health worldwide, with ovarian cancer (OC), cervical cancer (CC), and endometrial cancer (EC) being the most common. These cancers have distinct epidemiological characteristics, pathological mechanisms, and treatment strategies, but all significantly impact the female reproductive system and overall survival rates [1]. In recent years, advancements in molecular biology, immunotherapy, and genomics have led to breakthroughs in the diagnosis and treatment of OC, CC, and EC. However, early screening, personalized treatment, and drug resistance remain key research challenges [2].

Ovarian cancer has the highest mortality rate among gynecologic malignancies and is mainly classified into epithelial ovarian cancer, germ cell tumors, and sex cord-stromal tumors, with EOC being the most prevalent [3].Due to the lack of effective early screening methods, most patients are diagnosed at an advanced stage, leading to difficulties in treatment and low five-year survival rates. The current standard treatment includes surgical resection and platinum-based chemotherapy, but high recurrence rates remain a major challenge [4]. Recent studies have shown that the use of PARP inhibitors and immune checkpoint inhibitors can significantly improve survival in some patients [5]. Additionally, research has suggested that statin use may be associated with a reduced risk of ovarian cancer, providing new insights into potential prevention strategies [6].

Cervical cancer is primarily caused by persistent infection with high-risk human papillomavirus (HPV), which can lead to malignant transformation of cervical epithelial cells [7]. In recent years, the widespread administration of HPV vaccines has reduced the incidence of cervical cancer to some extent. However, vaccine coverage and efficacy vary by region, and cervical cancer remains a leading cause of cancer-related deaths among women in some low-income countries [8]. Moreover, serum metabolomic studies in cervical cancer patients have indicated that changes in specific metabolites may be associated with tumor progression, offering new directions for biomarker research [9].

Endometrial cancer is one of the most common gynecologic malignancies, with its incidence steadily increasing in recent years. It is primarily associated with obesity, diabetes, and hormone replacement therapy (Anderson et al., 2024). Endometrial cancer is mainly categorized into two types: Type I (estrogen-dependent) and Type II (non-estrogen-dependent), with Type II being more aggressive and having a poorer prognosis [10]. Currently, surgery remains the primary treatment for early-stage endometrial cancer, while targeted therapy and immunotherapy are emerging as promising approaches for advanced or recurrent cases [11]. A recent study found that polycystic ovary syndrome (PCOS) may increase the risk of endometrial cancer, providing a basis for screening high-risk populations [7].

The objective of this study is to systematically identify key pathogenic genes of EC, OC, and CC through the integration of multiple analytical.

methods and to explore their potential molecular mechanisms. These findings will help elucidate the shared and unique genetic foundations of these three gynecologic malignancies and provide scientific evidence for future biomarker development and precision medicine strategies.

## Methods

**Fig. 1:**
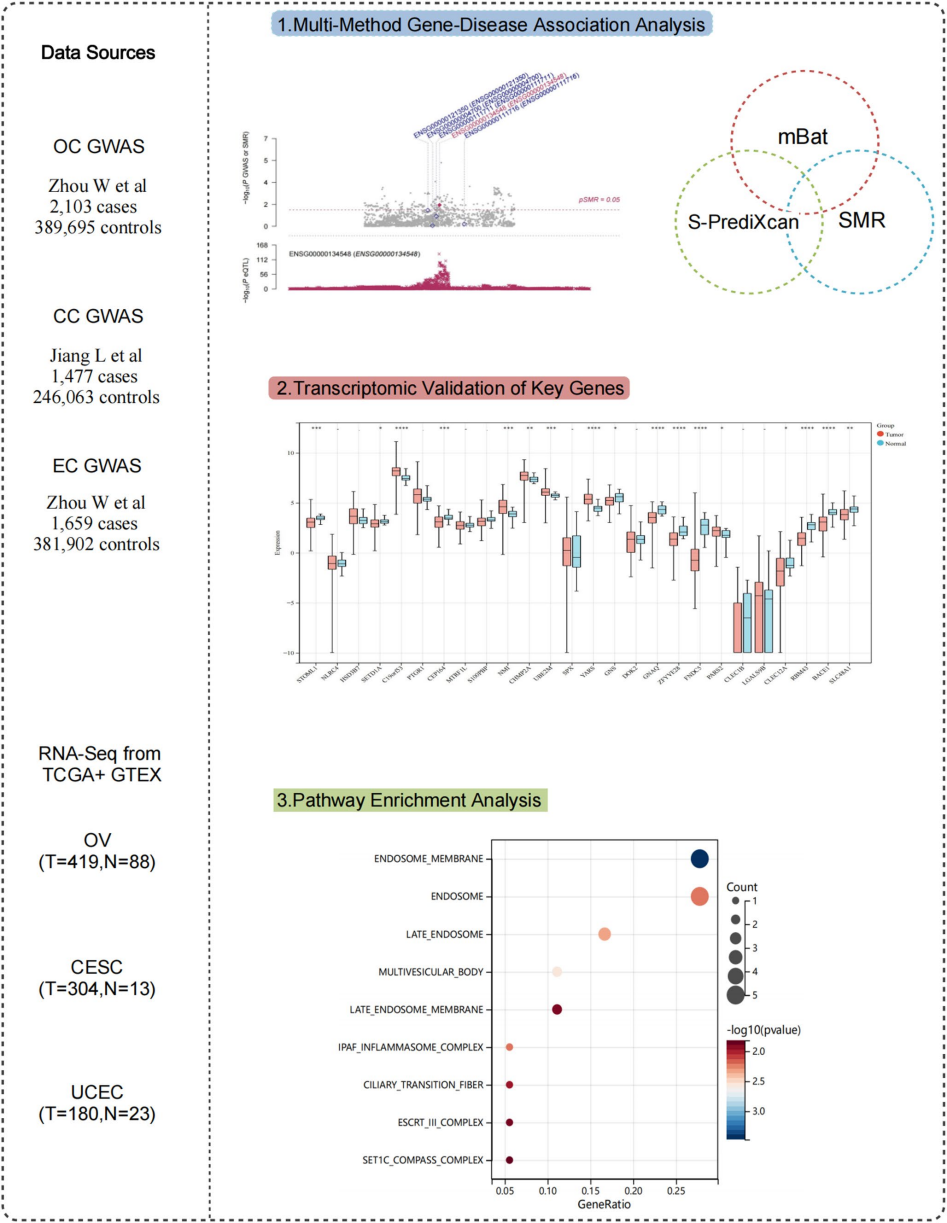
Flow chart. *OC* Ovarian cancer; *CC* Cervical cancer; *EC* Endometrial cancer; *CESC* Cervical squamous cell carcinoma and endocervical adenocarcinoma; *UCEC* Uterine corpus endometrial carcinoma

The research process is shown in Figure 1.

### Data Collection

In this study, summary statistics from genome-wide association studies (GWAS) on endometrial cancer (EC) were obtained from a recent large-scale meta-analysis by Jiang L et al. [12]. This meta-analysis, based on a European population, included 1,477 cases and 246,063 controls. Additionally, summary statistics for ovarian cancer (OC) and cervical cancer (CC) were obtained from a study by Zhou W et al. [13]. The OC dataset included 2,103 cases and 389,695 controls from a European population, while the CC dataset comprised 1,659 cases and 381,902 controls.

Blood eQTL data were sourced from the eQTLGen Consortium, which contains information on 10,317 single nucleotide polymorphisms (SNPs) associated with traits in 31,684 individuals [14].

### Integration of GWAS Genetic Association Signals and eQTL Data

The study employed the S-PrediXcan method to assess the association between gene expression levels and the target traits. Specifically, eQTL data from GTEx blood tissue were utilized to analyze key gene associations in OC, CC, and EC and to identify genes associated with these traits. S-PrediXcan predicts gene expression levels using GWAS summary statistics and evaluates their correlation with target traits. This method estimates the genetic component of gene expression at the gene level across different tissues and tests for associations with GWAS summary statistics at the SNP level [15]. A p-value < 0.05 was considered nominally significant.

### Mendelian Randomization Analysis Using Summary Data

The SMR software tool was developed to implement the SMR & HEIDI method. This approach integrates data from GWAS and expression quantitative trait locus (eQTL) studies to evaluate pleiotropic associations between gene expression levels and the traits of interest (OC, CC, and EC) [16]. Linkage disequilibrium (LD) calculations were based on the 1000 Genomes European reference data [17]. A *P* < 0.05 for SMR and a HEIDI heterogeneity test result >0.01 were used as inclusion criteria, indicating nominal significance.

### Powerful Gene-Trait Association Methods

This study used the gene-based association test mBAT-combo v1.94 for functional annotation. This method leverages GWAS summary data and is recognized for its superior ability to identify SNPs with masking effects. It combines GWAS summary data with linkage disequilibrium (LD) data from reference samples with individual genotype information to perform set-based association analyses for human complex traits. Traditional gene-based tests integrate SNP associations by summing chi-square statistics, but they may fail to detect SNPs with opposing effects due to their LD correlation. mBAT-combo is a novel set-based detection method with advantages in identifying masking-effect SNPs [18]. A P_mBATcombo < 0.05 was considered nominally significant for gene selection.

### RNA Sequencing Data Sources

We downloaded the standardized pan-cancer dataset TCGA TARGET GTEx (PANCAN, *N* = 19,131, G = 60,499) from UCSC (https://xenabrowser.net/). We then extracted expression data for 26 key genes identified in previous analyses and transformed each expression value using log2(x + 0.001). Subsequently, we selected samples related to cervical squamous cell carcinoma and adenocarcinoma (TCGA-CESC), endometrial carcinoma (TCGA-UCEC), and Ovarian Cancer (TCGA-OV). Using R software (version 3.6.4), we calculated expression differences between normal and tumor samples for each cancer type. Wilcoxon rank-sum and signed-rank tests were employed for significance analysis.

### Functional Enrichment Analysis

For gene set functional enrichment analysis, we first downloaded KEGG and GO (BP, CC, MF) subsets from [http://www.gsea-msigdb.org/gsea/downloads.jsp]. These genes were mapped to the respective background datasets, and enrichment analysis was conducted using the R package clusterProfiler (version 3.14.3) [19]. The minimum and maximum gene set sizes were set to 5 and 5000, respectively. A p-value < 0.05 and an FDR < 0.25 were considered statistically significant.

## Results

### Multilevel Analysis of Gene-Ovarian Cancer Association

**Fig. 2:**
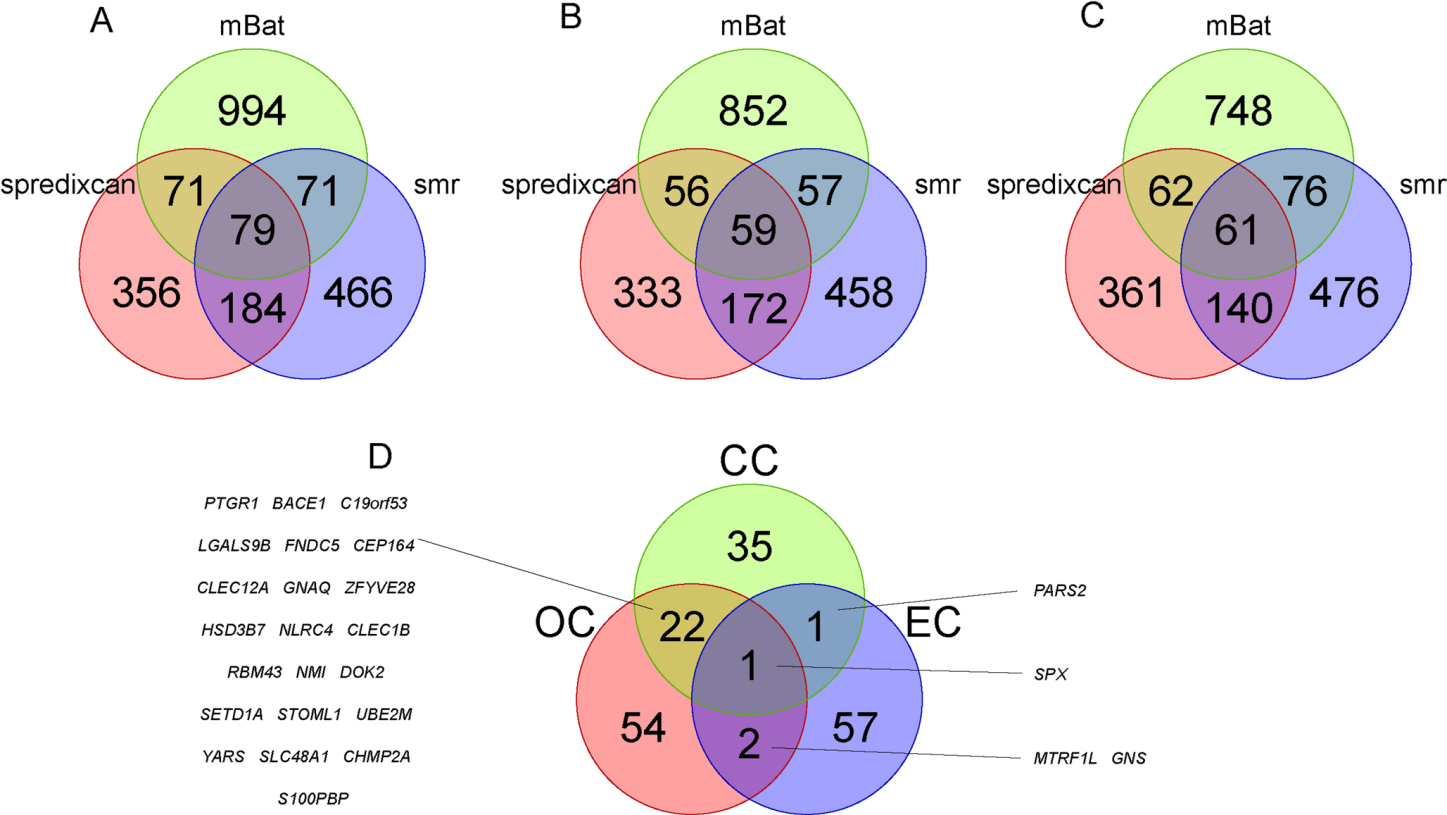
Venn diagrams showing the intersection of potentially associated genes identified by three analytical methods—S-PrediXcan, mBAT, and SMR. Panel **A** represents ovarian cancer, panel **B** represents cervical cancer, panel **C** represents endometrial cancer, and panel **D** displays the pleiotropic genes shared among ovarian cancer, cervical cancer, and endometrial cancer

We systematically evaluated the association between genes and ovarian cancer (OC) using various transcriptome-wide association methods. S-PrediXcan blood transcriptome analysis identified 690 genes with nominally significant associations (*p* < 0.05). The mBAT multi-gene joint test found 1,215 genes reaching the combined test significance threshold (P_mBATcombo < 0.05). The SMR integrative analysis identified 800 potential causal genes (p_SMR < 0.05 and p_HEIDI > 0.01). Notably, 79 key genes showed significant associations across all three independent analysis methods (Fig. 2A, Supplementary Tables 2–5), suggesting that these genes may have important biological significance in the pathogenesis of ovarian cancer.

### Multilevel Analysis of Gene-Cervical Cancer Association

We systematically evaluated the association between genes and cervical cancer (CC) using various transcriptome-wide association methods. S-PrediXcan blood transcriptome analysis identified 620 genes with nominally significant associations (*p* < 0.05). The mBAT multi-gene joint test found 1,024 genes reaching the combined test significance threshold (P_mBATcombo < 0.05). The SMR integrative analysis identified 746 potential causal genes (p_SMR < 0.05 and p_HEIDI > 0.01). Notably, 59 key genes showed significant associations across all three independent analysis methods (Fig. 2B, Supplementary Tables 6–9), suggesting that these genes may play a core regulatory role in the pathogenesis of cervical cancer.

### Multilevel Analysis of Gene-Endometrial Cancer Association

We systematically evaluated the association between genes and endometrial cancer (EC) using various transcriptome-wide association methods. S-PrediXcan blood transcriptome analysis identified 624 genes with nominally significant associations (*p* < 0.05). The mBAT multi-gene joint test found 947 genes reaching the combined test significance threshold (P_mBATcombo < 0.05). The SMR integrative analysis identified 753 potential causal genes (p_SMR < 0.05 and p_HEIDI > 0.01). Notably, 61 key genes showed significant associations across all three independent analysis methods (Fig. 2C, Supplementary Tables 10–13), suggesting that these genes may collaborate in the molecular regulatory network of endometrial cancer, providing important clues to its pathogenesis.

### Comorbidity Genes Identified in Genome-Wide Association Analyses

**Fig. 3:**
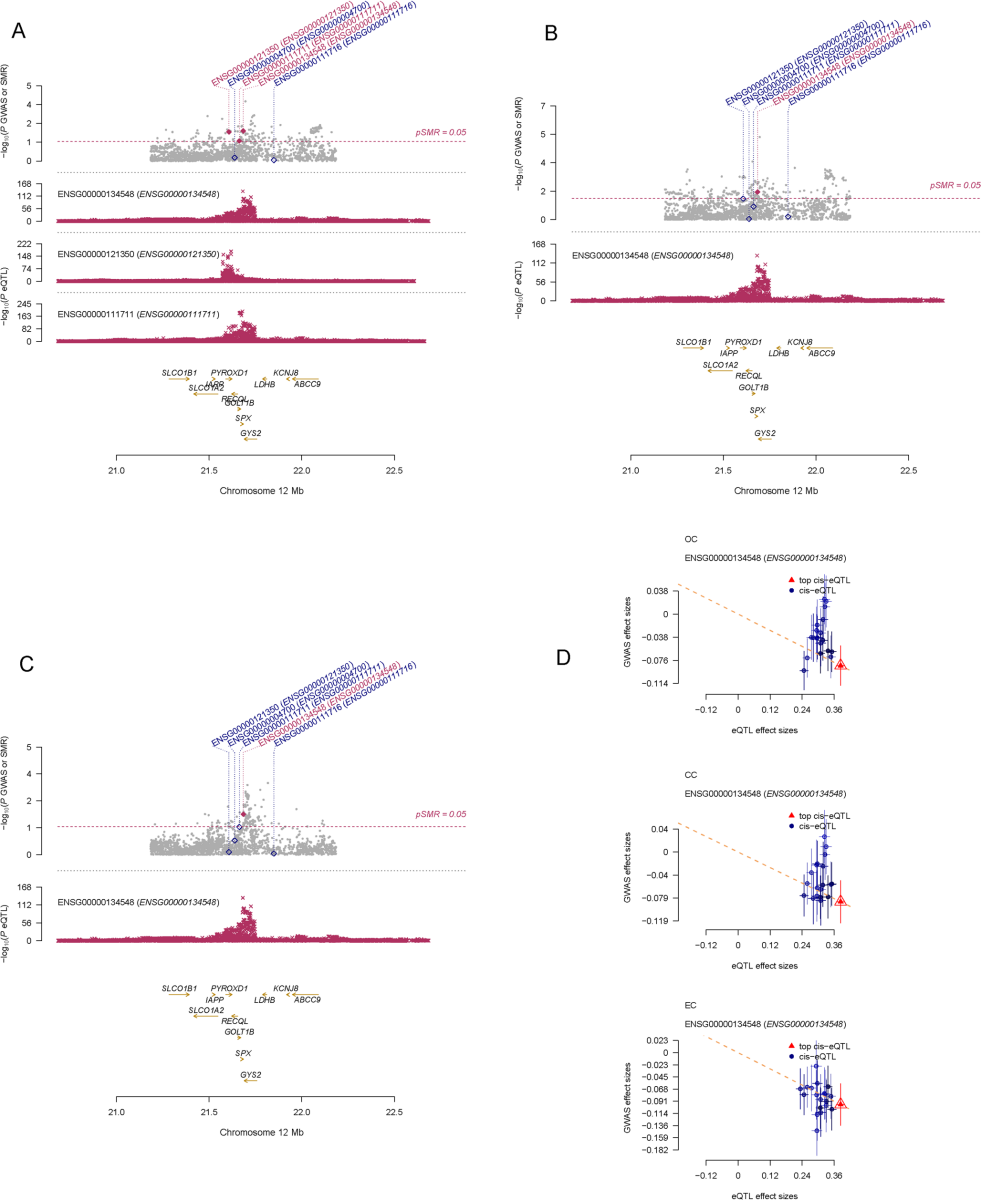
SMR analysis results of ENSG00000134548 (SPX) in relation to the three diseases. Panel **A** represents ovarian cancer (OC), panel **B** represents cervical cancer (CC), panel **C** represents endometrial cancer (EC), and panel **D** shows the scatter plot illustrating the relationship between SPX and the three diseases, where the slope of the diagonal line indicates the strength of the causal relationship

Based on the above analyses, we found that the gene SPX was associated with OC, CC, and EC. SPX was negatively correlated with all three diseases (Figs. 2D and 3). Additionally, we further identified comorbidity genes between diseases. OC and CC shared 22 comorbidity genes, including PTGR1, BACE1, C19orf53, LGALS9B, FNDC5, CEP164, CLEC12A, GNAQ, ZFYVE28, HSD3B7, NLRC4, CLEC1B, RBM43, NMI, DOK2, SETD1A, STOML1, UBE2M, YARS, SLC48A1, CHMP2A, and S100PBP. The comorbidity gene between CC and EC was PARS2, while the comorbidity genes between OC and EC were MTRF1L and GNS.

### Differential Transcriptomic Analysis

**Fig. 4:**
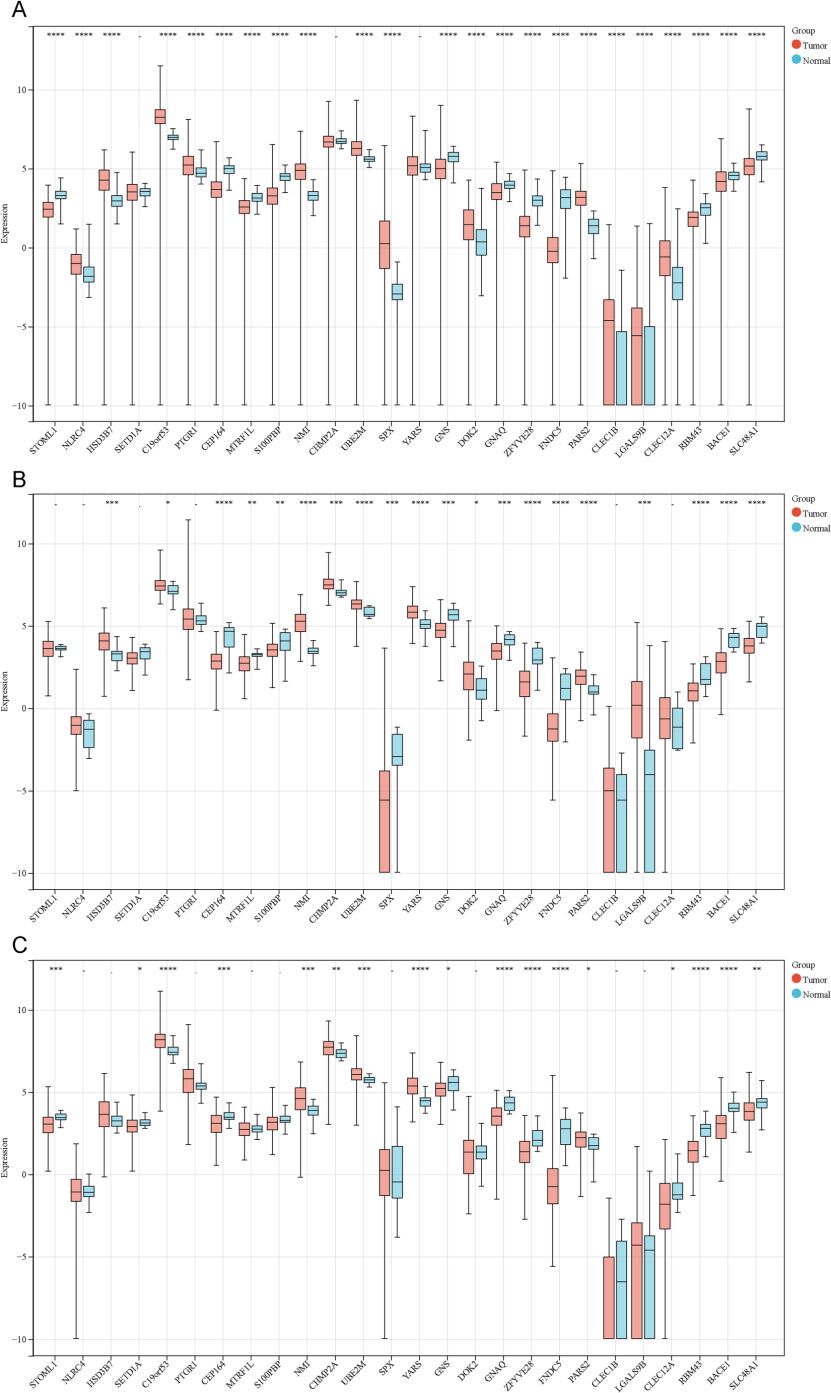
Box plots showing the expression differences of 26 key pleiotropic genes between tumor and normal groups in transcriptomic data. Panel **A** represents ovarian cancer (OC), panel **B** represents cervical squamous cell carcinoma and endocervical adenocarcinoma (CESC), and panel **C** represents uterine corpus endometrial carcinoma (UCEC)

Given the 26 key genes identified in the above analyses, we conducted differential transcriptomic analysis between tumor and normal control groups. Among them, 23 genes showed significant differential expression between Ovarian Cancer (OC) and the control group, with 12 genes upregulated and 11 downregulated in the tumor group (Fig. 4A, Supplementary Table 14). In cervical squamous and adenocarcinoma (CESC), 20 genes showed significant differential expression, with 17 genes upregulated and 3 downregulated in the tumor group (Fig. 4B, Supplementary Table 15). In endometrial carcinoma (UCEC), 17 genes showed significant differential expression, with 6 genes upregulated and 11 downregulated in the tumor group (Fig. 4C, Supplementary Table 16). Based on blood eQTL results from the SMR analysis, SPX was negatively correlated with both OC and CC, suggesting a potential protective role in the development of these diseases. However, differential transcriptomic analysis showed that SPX was significantly upregulated in the OC group and significantly downregulated in the CESC group compared to their respective control groups. These differences in results may reflect the heterogeneous regulation of SPX in different tissue environments or pathological states.

### Functional Enrichment Analysis

**Fig. 5:**
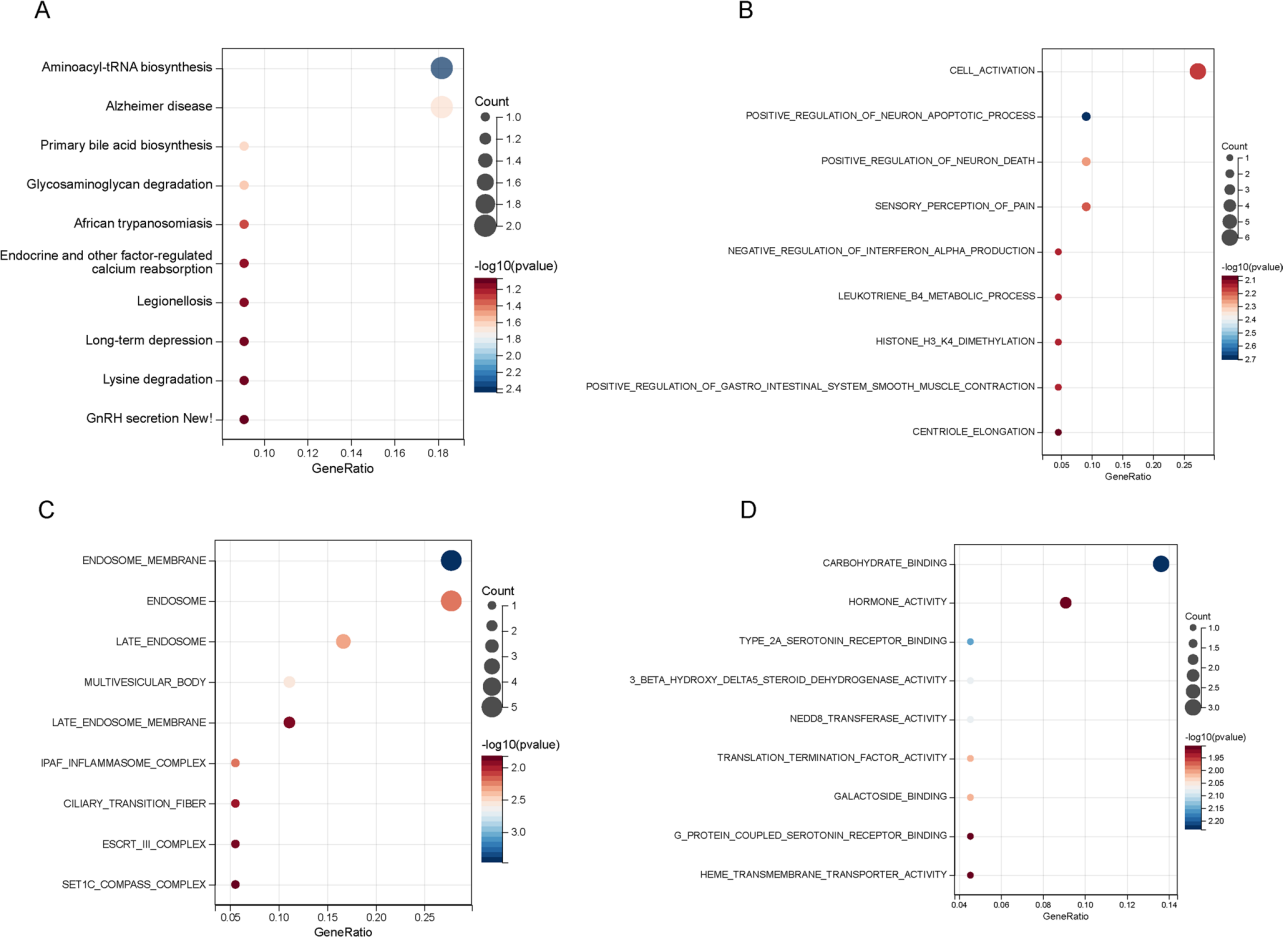
Enrichment analysis of 26 key genes. Panel **A** represents KEGG pathways, panel **B** represents GO biological processes (BP), panel **C** represents GO cellular components (CC), and panel **D** represents GO molecular functions (MF)

KEGG analysis indicated that these pathways might involve protein synthesis (hsa00970), neurological diseases (hsa05010), lipid metabolism (hsa00120), and carbohydrate metabolism (hsa00531) (Fig. 5A, Supplementary Table 17). The GO biological process (BP) enrichment analysis mainly involved processes such as regulation of neuron apoptosis, neuronal death, pain perception, cell activation, interferon regulation, leukotriene metabolism, and histone methylation (Fig. 5B, Supplementary Table 18). The GO cellular component (CC) enrichment analysis primarily involved organelles related to endosomes, inflammatory complexes, and ciliary structures (Fig. 5C, Supplementary Table 19). The GO molecular function (MF) enrichment analysis mainly involved carbohydrate binding, neurotransmitter receptor binding, steroid metabolism, ubiquitin-related modifications, and translation termination, which may be related to cell signal transduction, metabolic regulation, and protein modifications (Fig. 5D, Supplementary Table 20). In summary, the comorbidity genes of OC, CC, and EC may influence tumor development through various biological pathways and functions, including protein synthesis, lipid and carbohydrate metabolism, neuronal regulation, inflammation, cell signal transduction, and protein modification.

## Discussion

This study systematically analyzed the comorbidity genes and their potential molecular mechanisms in endometrial cancer (EC), ovarian cancer (OC), and cervical cancer (CC) by integrating multi-omics data. Through GWAS, eQTL, and transcriptomic analyses, we identified key genes, such as SPX, and their complex regulatory patterns across these three cancers. We also found that metabolic reprogramming, neuro-regulation, and epigenetic modifications may constitute a common pathological foundation for these cancers.

By employing multi-method integration strategies, such as S-PrediXcan, SMR, and mBAT-combo, we effectively overcame the limitations of single-method approaches. Among them, mBAT-combo, which integrates SNP effect directions, significantly improved the detection capability for masking-effect genes [18]. This advantage aligns with recent studies that highlight the value of multi-method integration in complex trait analysis [20]. Notably, the SPX gene exhibited a negative correlation with OC and CC in SMR analysis, but its expression direction in tumor tissues showed disease-specific differences. This inconsistency may reflect the regulatory heterogeneity between blood eQTL and the tumor microenvironment: blood eQTL mainly captures the genetic baseline expression levels of genes [14], while gene expression in tumor tissues may be driven by intracellular mutations or epigenetic reprogramming [21]. Genetic influences on expression are often tissue-specific; indeed, GTEx and other studies have demonstrated that certain eQTLs may even reverse their direction across tissues, likely due to differences in epigenetic states [22]. Moreover, tumor cells reside within a unique microenvironment—characterized by altered hormonal signaling, hypoxia, inflammation, and stromal interactions—that reshapes transcription through epigenetic mechanisms [23, 24]. Consequently, a protective genetic signal observed in blood may not translate into the same expression pattern within tumor tissues, which could account for the opposing SPX trends identified in ovarian and cervical cancers.Similar tissue-specific regulatory phenomena have also been reported in tumor suppressor genes, such as TP53 [25].

The role of SPX in female reproductive system cancers has been gradually gaining attention, especially in OC, where studies have suggested that it may regulate tumor cell phosphate metabolism through XPR1/SLC53A1, thereby promoting tumor cell proliferation and potentially serving as a therapeutic target [26]. Additionally, high SPX expression may be associated with poor prognosis in high-grade serous OC patients [27]. In EC, although relevant research is scarce, literature has indicated that SPX may participate in metabolic regulation in cancer cells [28]. Overall, SPX’s role in OC is relatively well-established, while its specific function in EC and CC still requires further research and validation.

Our study identified several comorbidity genes shared between EC, OC, and CC, including PTGR1, BACE1, SETD1A, and UBE2M. Functional enrichment analysis revealed that these genes are primarily involved in protein synthesis, lipid metabolism, carbohydrate metabolism, neurodegenerative diseases, neuronal apoptosis regulation, inflammatory responses, and epigenetic modifications. GO analysis highlighted the enrichment of neuronal apoptosis regulation and pain perception pathways, supporting the importance of neuroinfiltration in the development of gynecological cancers. Furthermore, research has shown that SPX is expressed in both zebrafish and mammalian brains and spinal cords, potentially affecting the development of neural circuits [29]. As a neuropeptide, SPX is widely expressed in multiple regions of the nervous system and plays crucial roles in energy metabolism, anxiety regulation, and neurodevelopment [30–33].

Among the comorbidity genes of OC and CC, FNDC5 may play a tumor-suppressive role in both cancers, primarily mediated through the PI3K/AKT signaling pathway [34–36]. SETD1A may promote the progression of OC and affect the sensitivity of HR-deficient tumors to PARP inhibitors [37, 38]. GNAQ and ZFYVE28 may be involved in the plasticity and metabolic regulation of cancer cells [39, 40]. NLRC4 inflammasome may have a role in immune regulation in CC and OC, although related research is still limited [41–44]. Additionally, S100PBP may serve as a potential therapeutic target for CC [45].

Previous studies have revealed the roles of these genes in individual cancer types. BACE1-AS regulates BACE1 to promote tumor growth and migration in ovarian cancer stem cells [46]. NMI is consistently overexpressed in both cervical and ovarian cancers and demonstrates strong diagnostic and prognostic value [47]. Reduced expression of DOK2 is closely associated with cisplatin resistance in ovarian cancer [48]. In addition, CLEC12A is a key marker of tumor-infiltrating immune cells in cervical cancer and reflects the state of the tumor microenvironment [49].

Importantly, our study further demonstrates that these four genes are not restricted to a single cancer type but are simultaneously associated with both OC and CC. This finding suggests that they may represent critical cross-cancer molecular pathways, deepening our understanding of shared mechanisms in gynecological malignancies and expanding their potential clinical relevance. Specifically, BACE1-AS and DOK2 are strongly related to chemotherapy sensitivity and may serve as predictors of treatment response or targets for overcoming resistance; NMI shows particular promise in molecular subtyping and prognostic assessment; and CLEC12A offers new perspectives for investigating the tumor immune microenvironment and responses to immunotherapy.

Despite significant progress, this study has several limitations. First, our analyses were based primarily on GWAS data from European populations, and future studies with sufficiently powered GWAS in non-European populations, particularly East Asians, will be important to validate these findings. The effects of comorbidity genes may shift significantly in these populations. Second, differences in blood and tissue expression still require in-depth analysis through spatial transcriptomics and single-cell sequencing techniques, especially the specific regulatory networks of tumor-associated fibroblasts and immune cells.Third, due to data availability and sample size limitations, we did not stratify cervical cancer (HPV-dependent and HPV-independent), endometrial cancer, or ovarian cancer by histological subtypes. Our study primarily focused on cross-disease overall associations, and future research could further explore subtype-specific mechanisms based on these findings. Moreover, although newly discovered genes like CLEC12A and RBM43 are related to innate immunity and RNA splicing in functional enrichment analysis, their specific roles in tumorigenesis still need validation through CRISPR screening and organoid models. Future research should focus on constructing the metabolic-neuro-regulatory network map of SPX and explore the clinical translation pathways of new targets, such as S100PBP, ultimately bridging the gap from mechanistic research to precision medicine.

## Conclusion

This study systematically identified comorbidity genes in EC, OC, and CC by integrating multi-omics data and revealed their potential molecular mechanisms. We found complex regulatory patterns of key genes, such as SPX, in these three cancers, and that metabolic reprogramming, neuro-regulation, and epigenetic modifications may constitute their shared pathological foundation. Additionally, several comorbidity genes play crucial roles in neuroinfiltration, immune regulation, and tumor metabolism. Despite the limitations regarding population applicability and mechanistic validation, our findings provide new molecular targets and research directions for the precise diagnosis and treatment of gynecological cancers.

## Supplementary Information

Below is the link to the electronic supplementary material.

Supplementary Material 1 (XLS 2.75 MB)

## Data Availability

This study utilized GWAS data from the GWAS Catalog. mBat: https://yanglab.westlake.edu.cn/software/gcta/#mBAT-combo. S-PrediXcan: https://github.com/hakyimlab/MetaXcan. SMR: https://yanglab.westlake.edu.cn/software/smr/#Overview. EQTL: https://eqtlgen.org/cis-eqtls.html. The transcriptomic data were obtained from https://xenabrowser.net/.
